# To Give a Smooth, Finished Surface to Vulcanite

**Published:** 1900-04-15

**Authors:** B. J. Cigrand


To Give a Smooth, Finished Surface to Vulcanite.—After investing in the flask, remove the wax absolutely and paint the palatal and lingual surfaces with a mixture of liquid glass and silver bronze. When hard, cover with soapsuds to prevent adhesion to the vulcanite. The case will come out smooth, with a glistening appearance.—*B. J. Cigrand, Dental Digest.*

